# Correction to “The knockdown of lncRNA DLGAP1‐AS2 suppresses osteosarcoma progression by inhibiting aerobic glycolysis via the miR‐451a/HK2 axis”

**DOI:** 10.1111/cas.16403

**Published:** 2024-11-12

Zheng C, Li R, Zheng S, Fang H, Xu M, Zhong L. The knockdown of lncRNA DLGAP1‐AS2 suppresses osteosarcoma progression by inhibiting aerobic glycolysis via the miR‐451a/HK2 axis. *Cancer Sci* 2023;114(12):4747–4762.

We used the incorrect U6 prime sequence in this study. For the validity and rigorousness of research results, we redesigned the correct U6 primer sequence and repeated the related experiments (Figures 4, 5 and 6). Please kindly change the data of Figures 4D, 4E, and 6F, as well as U6 prime sequence U6 (F: 5′‐CTCGCTTCGGCAGCACAT‐3′, R: 5′‐TTTGCGTGTCATCCTTGCG‐3′) in manuscript.

The corrected Figures 4D, 4E, and 6F is as follows:

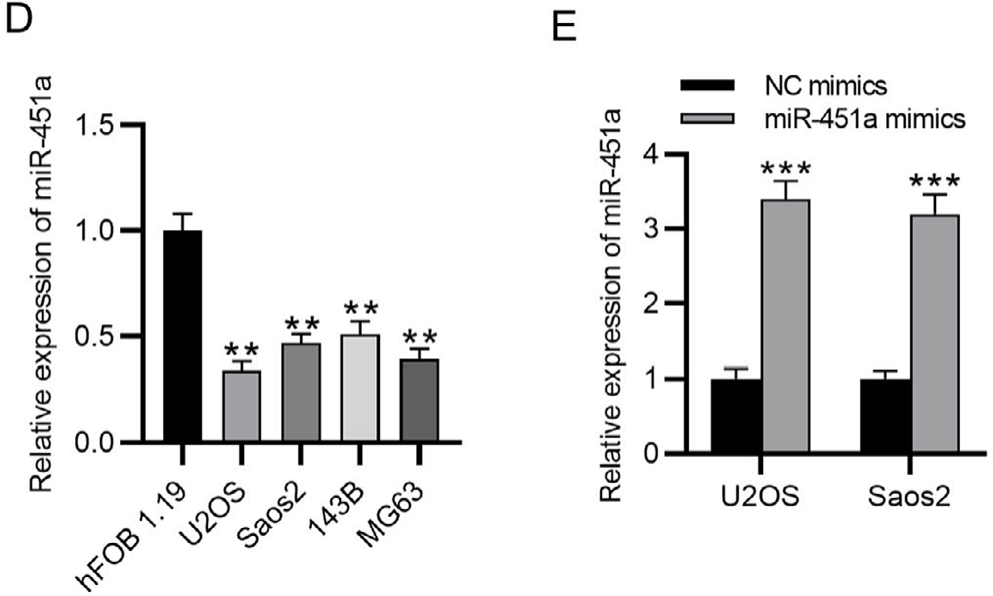

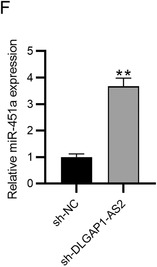

We apologize for this error.

